# Supplementary material for: Mastocytosis-derived extracellular vesicles deliver miR-23a and miR-30a into pre-osteoblasts and prevent osteoblastogenesis and bone formation
Source: Nat Commun. 2021 May 5;12:2527. doi: 10.1038/s41467-021-22754-4 (PMC8100305; doi:10.1038/s41467-021-22754-4)
Supplement: Supplementary file 1 — Supplementary Information [file 41467_2021_22754_MOESM1_ESM.pdf]

Mastocytosis-derived extracellular vesicles deliver miR-23a and miR-30a into pre-osteoblasts and prevent osteoblastogenesis and bone formation

Do-Kyun Kim, Geethani Bandara, Young-Eun Cho, Hirsh D. Komarow, Danielle R. Donahue, Baktiar Karim, Moon-Chang Baek, Ho Min Kim, Dean D. Metcalfe, Ana Olivera*

***Correspondence:**
Ana Olivera
ana.olivera@nih.gov

Supplementary material contents:

Supplementary Fig. 1
Supplementary Fig. 2
Supplementary Fig. 3
Supplementary Fig. 4
Supplementary Fig. 5
Supplementary Fig. 6
Supplementary Fig. 7
Supplementary Fig. 8
Supplementary Fig. 9

Supplementary Table 1
Supplementary Table 2
Supplementary Table 3
Supplementary Table 4
Supplementary References

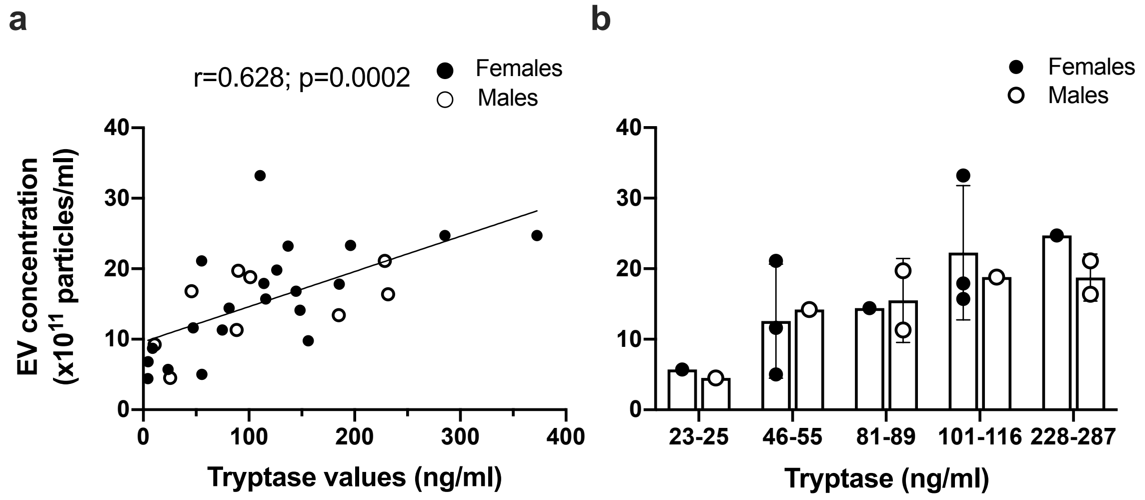

Supplementary Fig. 1- Gender and serum concentrations of EVs in SM. **a** Pearson's correlation between extracellular vesicle (EV) concentration in serum and tryptase values for each patient, identifying female (black circles) or male subjects (open circles). **b** Bar graph illustrating similar serum EV concentrations in female and male patients within close tryptase level ranges. The circles indicate the EV concentration values from each patient with the indicated ranges of serum tryptase values (n=1 for males or females in the 23-25 ng/ml tryptase range; n=3 females and n=1 male in the 46-55 ng/ml range; n=1 female and n=2 males in the 81-89 ng/ml range; n=3 females and n=1 male in the 101-116 ng/ml range; and n=1 female and n=2 males in the 228-287 ng/ml range). Data are the mean ± SD when applicable.

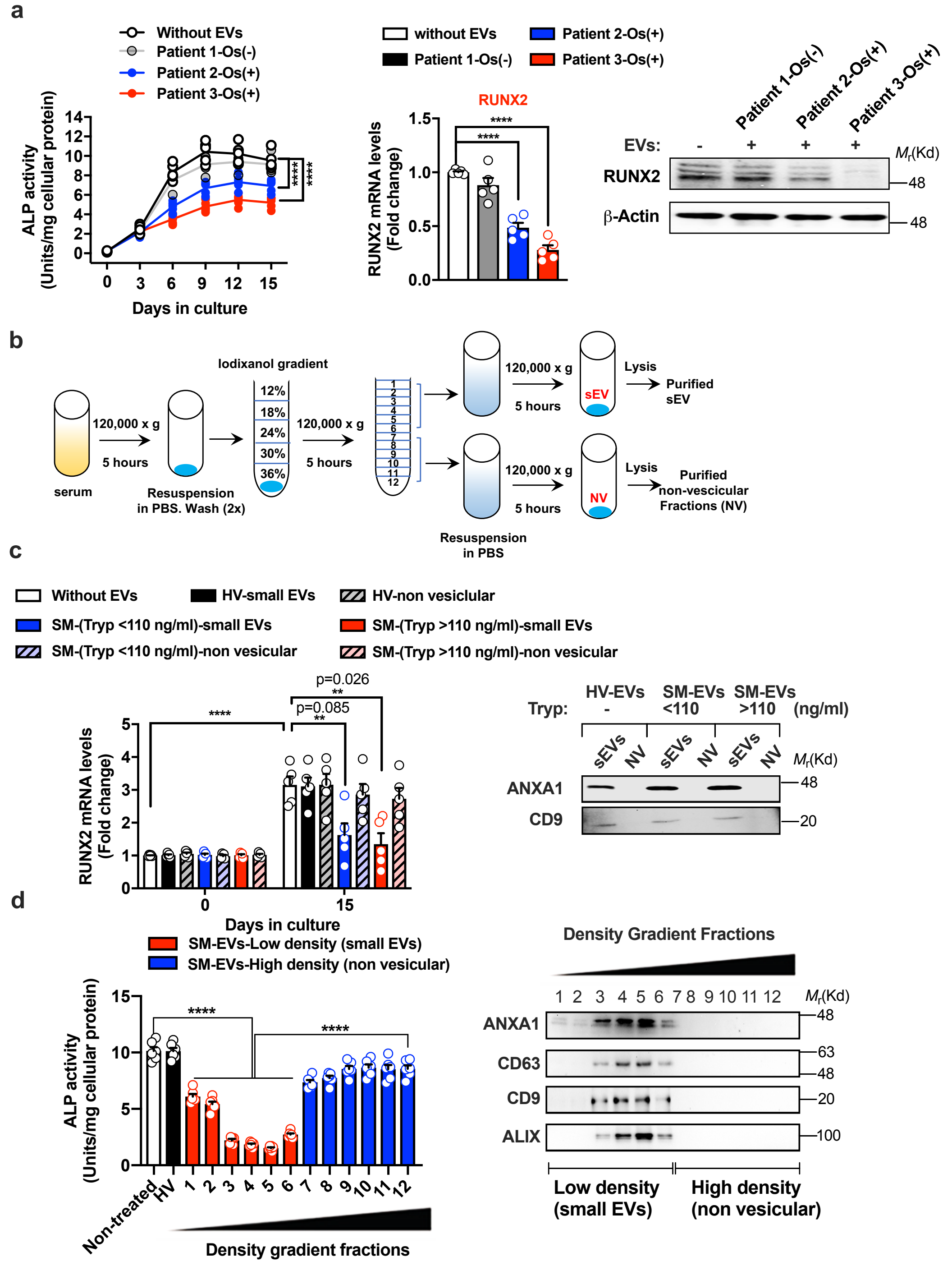

Supplementary Fig. 2-SM-EVs from BM, similar to those from serum, attenuate osteoblast differentiation; and the effect on osteoblasts is independent on the isolation method of EVs. a Effects of bone marrow (BM)-derived extracellular vesicles (EVs) from 3 patients with systemic mastocytosis (SM) on the differentiation of hFOB1.19 osteoblasts, measured as alkaline phosphatase (ALP) activity released to the culture media, and expression of RUNX2 in cells. Data represent mean ± SEM of a representative experiment (n=5 biological replicates), repeated at least once with EVs isolated from the same patients. A two-way ANOVA was used for statistic comparison of the curves (compared to untreated). Individual

data points are shown. Unpaired Student t-tests (two-tailed) were used for the right panel. ****$p<0.0001$. Patient 1 had ISM (age 44; White; tryptase=241 ng/mL) with no osteopenia/osteoporosis (Patient 1-Os(-)); patient 2 had ASM (age 66; White; tryptase=262 ng/mL) with osteopenia (Patient 2-Os(+)); and patient 3 had ISM (age 42; Asian; tryptase=18 ng/mL) with osteopenia and osteoporosis (Patient 3-Os(+)). All were D816V positive. **b** Representation of the protocol of isolation of serum EVs by density gradient ultracentrifugation. **c** Effects of pooled fractions corresponding to small EVs (sEVs) or non-vesicular (NV) fractions (as defined in **b**) on the differentiation of hFOB1.19 osteoblasts measured by the expression of RUNX2 by qRT-PCR (n=5 biological replicates). Data were normalized to GAPDH expression levels and expressed as fold change compared to untreated cells (white bars) immediately after plating (at day 0). EVs were isolated from all healthy volunteers and pooled (HV-EVs; back bars and hatched bars for sEVs and NV fractions respectively as indicated in the figure); patients with SM and tryptase values lower than the median (SM-EVs (Tryp<110 ng/mL); blue solid bars for sEVs and hatched bars for NV fractions) or SM-EVs with tryptase values greater than the median (SM-EVs (Tryp>110 ng/mL); red solid bars for sEVs and hatched bars for NV fractions). Presence of the small EV markers annexin A1 (ANXA1) and CD9 in sEVs and their absence in the NV pool is demonstrated by Western blotting on the right panel. **d** Effects of individual fractions (obtained as indicated in **b**) on the inhibition of ALP activity (n=6 biological replicates) in a representative experiment. Expression of the indicated markers of EVs is shown on the right panel. Data in **c** and **d** represent mean ± SEM. Statistical comparisons were performed using unpaired Student t-tests (two-tailed). **$p<0.01$; and ****$p<0.0001$.

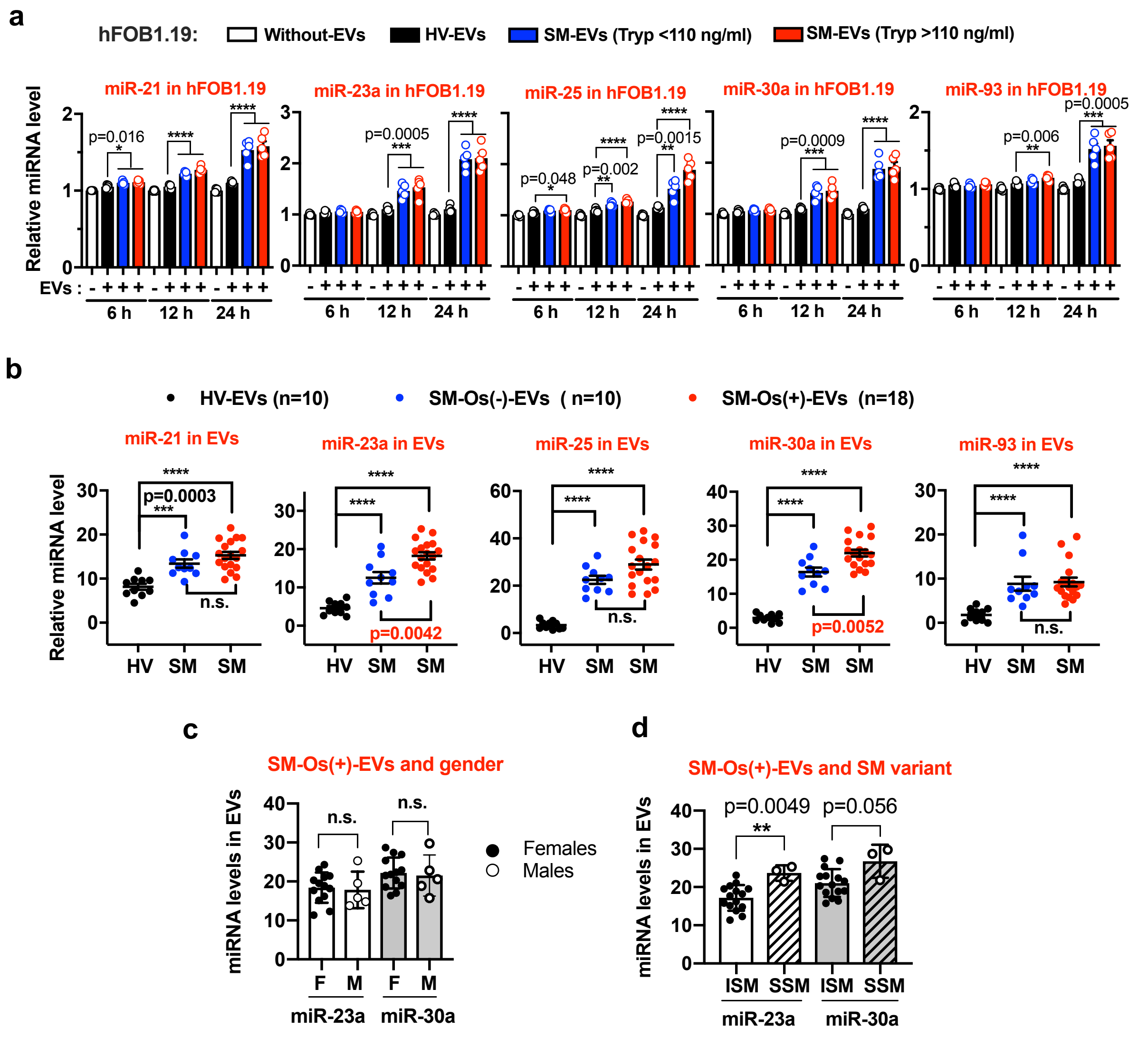

Supplementary Fig. 3- Early transfer of miRNAs from SM-EVs into osteoblasts and miRNAs levels in SM-EVs depending on bone disease, gender and SM variant. **a** Levels of miR-21, mR-23a, miR-25, miR-30a and miR-93 in hFOB1.19 osteoblasts after treatment or not (white bars) with EVs isolated from all healthy volunteers (HV-EVs; back bars), patients with systemic mastocytosis (SM) and tryptase values lower than the median (SM-EVs (Tryp<110 ng/mL); blue bars), or SM-EVs with tryptase values greater than the median (SM-EVs (Tryp>110 ng/mL); red bars). hFOB1.19 osteoblasts were plated and treated once with 100 µg of a pool of EVs from each subgroup in Group 1 of Supplementary Table 1 for 6, 12 or 24 h. RNA was isolated from cells and equal amounts of RNA reversed-transcribed. MiRNA levels were determined by qRT-PCR and normalized to GAPDH. Data is expressed as fold change in comparison to untreated cells each day and are the mean ± SEM (n=5 biological replicates) of a representative experiment, repeated at least twice with similar results. *p<0.05; **p<0.01; ***p<0.001; and ****p<0.001 using unpaired Student t-test (two-tailed). **b** Levels of miR-21, miR-23a, miR-25, miR-30a and miR-93 isolated from 100 μg of HV-EVs (n=10

individuals), patients with SM with osteoporosis/osteopenia (red circles; SM-Os(+)-EVs, n=18) or without osteoporosis/osteopenia (blue circles; SM-Os(-)-EVs, n=10). The data includes EVs from patients described in Group 1 of Supplementary Table 1 and shown in Fig. 4a but regrouped to emphasize differences between patients with or without diagnosed osteoporosis/osteopenia, plus 10 patients described in Group 2 of Supplementary Table 1. The expression levels of the miRNA species were measured by qRT-PCR from equal amounts of total RNA and normalized to the levels of a spike-in control (miR-39). A Mann-Whitney test (two-tailed) was used for statistical comparisons between groups. ***p<0.01;****p<0.0001 or as indicated in the figure. Data represent the mean ± SEM. **c** Levels of miR-23a and miR-30a, measured as in **b**, in female (F; n=13) and male (M; n=5) patients from the group of patients with SM with osteoporosis/osteopenia shown in **b** in red circles. n.s., not significant using Mann-Whitney test (two-tailed). Data represent the mean ± SD. The levels of these mi-RNAs were also not different between genders when considering all patients in b (with and without osteoporosis/osteopenia); miR-23a levels were 16.0±5.1 (mean ± SD) for females and 16.4±4.2 (mean ± SD) for males; and miR-30a levels were 20.0±4.6 (mean ± SD) for females and 19.3±5.2 (mean ± SD) for males. **d** Levels of miR-23a and miR-30a in patients with SM with osteoporosis/osteopenia shown in **b** in red circles separated by disease variant: ISM, indolent SM (n=15 patients); SSM, smoldering SM (n=3 patients). **p<0.01 using a Mann-Whitney test (two-tailed). Data represent the mean ± SD.

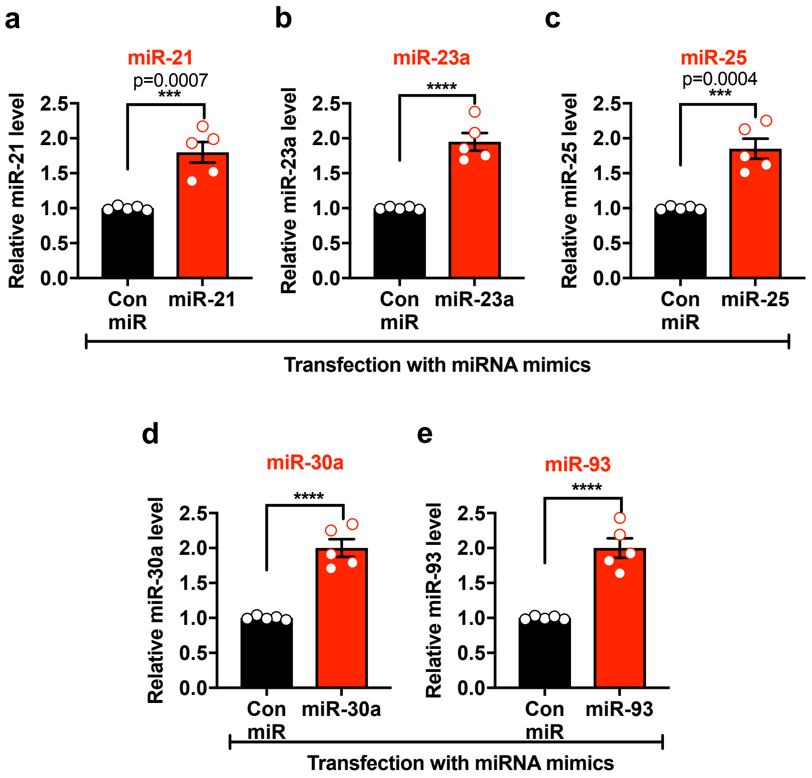

Supplementary Fig. 4- Increases in the levels of miRNAs in hFOB1.19 pre-osteoblasts after transfection with miRNA mimics. Expression levels in hFOB1.19 osteoblastic cells of miR-21 (**a**), mR-23a (**b**), miR-25 (**c**), miR-30a (**d**) and miR-93 (**e**) 6 d after transfection with 100 nM of the respective miRNA mimics or control miRNA (Con miR). hFOB1.19 cells were plated at 39.5ºC and next day transfected with the miRNA mimics. The transfection media was replaced after 2 d and 4 d later and cellular RNA was extracted. Equal amounts of RNA were reversed transcribed and the levels of the miRNA species were determined by qRT-PCR and normalized to the levels of GAPDH. Data are expressed as fold change compared to cells transfected with miRNA mimic negative control (Con miR). Data represent the mean ± SEM (n=5 biological replicates) of a representative experiment, repeated at least twice with similar results. ***p<0.001; ****p<0.0001 using an unpaired Student-t test (two-tailed).

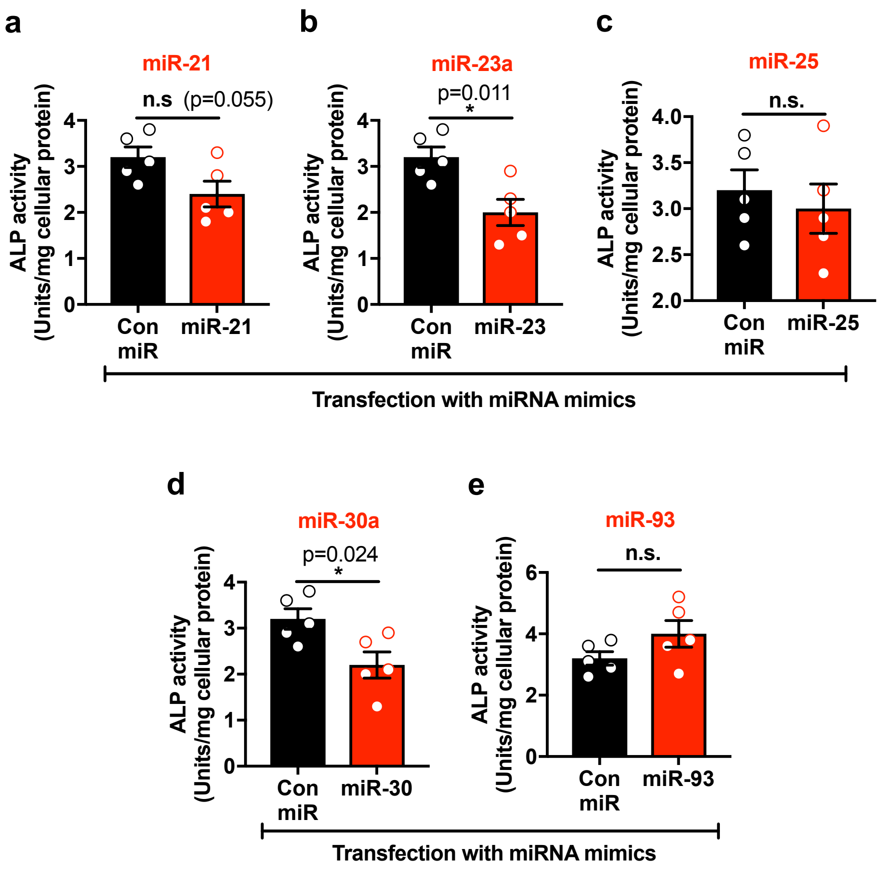

Supplementary Fig. 5-MiR-21, miR-23a and miR-30a, reduce ALP activity a day after transfection. Effect of miRNA mimics for miR-21 (**a**), mR-23a (**b**), miR-25 (**c**), miR-30a (**d**) and miR-93 (**e**) on osteoblast differentiation. hFOB1.19 osteoblasts were plated at 39.5°C and transfected with the indicated miRNA mimics or control miRNA (Con miR). After 2 d, the transfection media was changed and alkaline phosphatase (ALP) activity in the supernatants measured 1 d later. ALP activity was corrected by the amount to total protein in the cell lysates. Data represent the mean ± SEM (n=5 biological replicates) of a representative experiment, repeated at least twice with similar results. *p<0.05; n.s., not significant using unpaired Student-t tests (two-tailed).

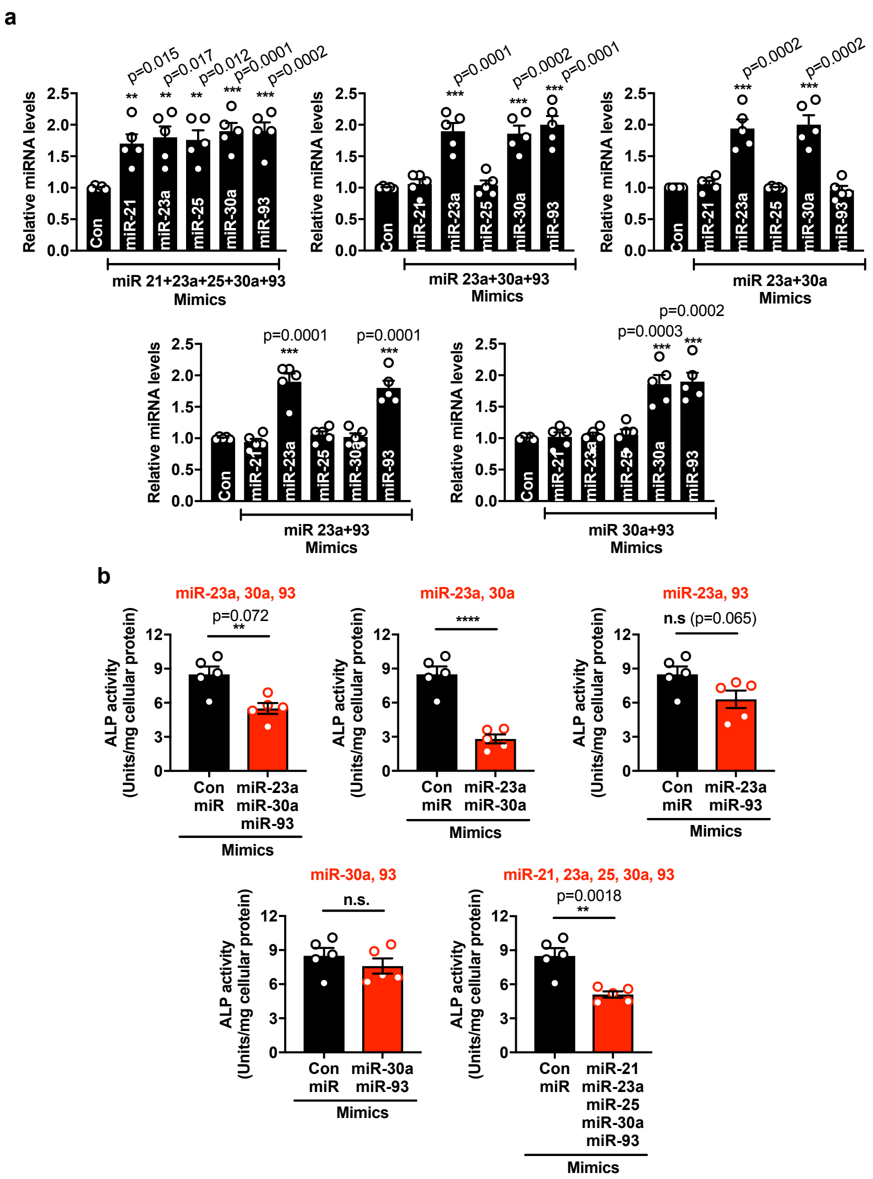

Supplementary Fig. 6-Transfection with a combination of miR-23a and miR-30a mimics causes greater effects on osteoblast differentiation than each individually. a-b Effect of combinations of miRNA mimics on the levels of the corresponding miRNA species in osteoblasts (**a**) and in alkaline phosphatase (ALP) activity (**b**). hFOB1.19 osteoblasts were plated and next day transfected with the indicated combinations of mimics. After 2 days media was replaced, and RNA was extracted from the cells after 5 additional days to measure the levels of the corresponding miRNAs (**a**) and the culture media to measure ALP activity (**b**). The levels of the miRNA species from equal amounts of total RNA were determined by qRT-PCR and normalized to the levels of GAPDH. ALP activity was corrected by the amount to total protein in the cell lysates. Data in **a** and **b** represent the mean ± SEM (n=5 biological replicates) of a representative experiment, repeated at least twice with similar results. n.s., not significant; **p<0.01; ***p<0.001; ***p<0.0001 using an unpaired Student t-test (two-tailed).

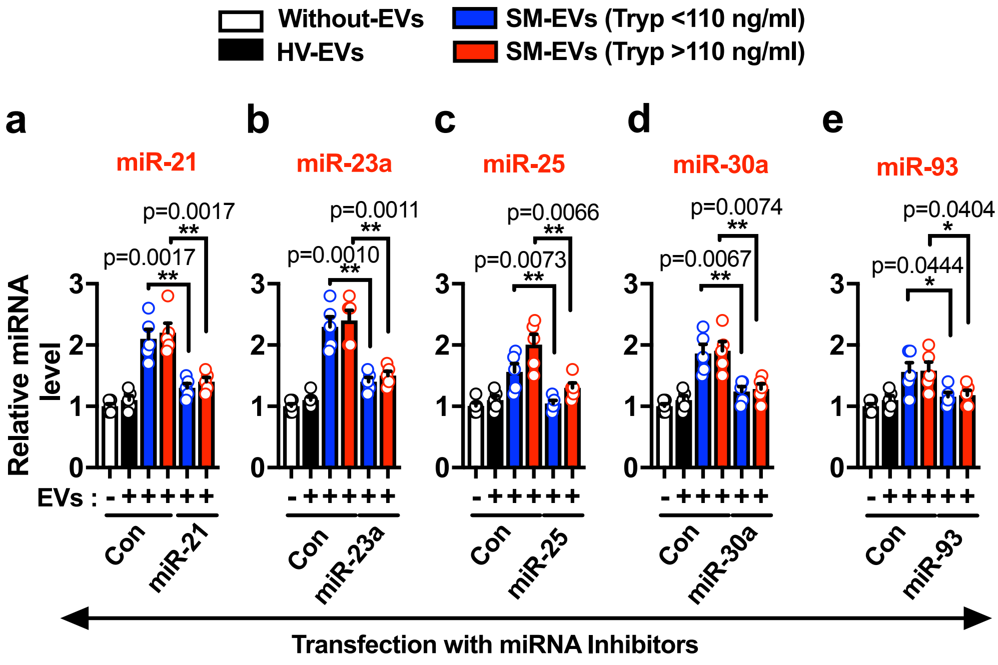

Supplementary Fig. 7- Knockdown of miR-21, mR-23a, miR-25, miR-30a and miR-93 prevent the increase in levels of the respective miRNAs in osteoblasts after treatment with of SM-EVs. Expression levels of miR-21 (**a**), mR-23a (**b**), miR-25 (**c**), miR-30a (**d**) and miR-93 (**e**) 2 d after transfection with 100 nM of the respective miRNA inhibitors in hFOB1.19 cells treated or not (white bars) with EVs isolated from all healthy volunteers (HV-EVs; back bars), patients with systemic mastocytosis (SM) and tryptase values lower than the median (SM-EVs (Tryp<110 ng/mL); blue bars), or SM-EVs with tryptase values greater than the median (SM-EVs (Tryp>110 ng/mL); red bars). hFOB1.19 cells were seeded and then treated with the indicated pool of EVs (100 μg) for a total of 7 d. Cells were then transfected with control miRNA (Con) (first four bars) or the indicated miRNA inhibitors (last two bars) for 2 d. Transfection media was replaced and 5 d later, cellular RNA was extracted reversed transcribed and the levels of the miRNA species (from equal amounts of total RNA) were determined by qRT-PCR. Data in **a-e** are expressed as fold change compared to untreated cells transfected with control miRNA (Con) and are the mean ± SEM (n=5 biological replicates) of a representative experiment, repeated at least twice with similar results. *p<0.05;**p<0.01 using an unpaired Student t-test (two-tailed).

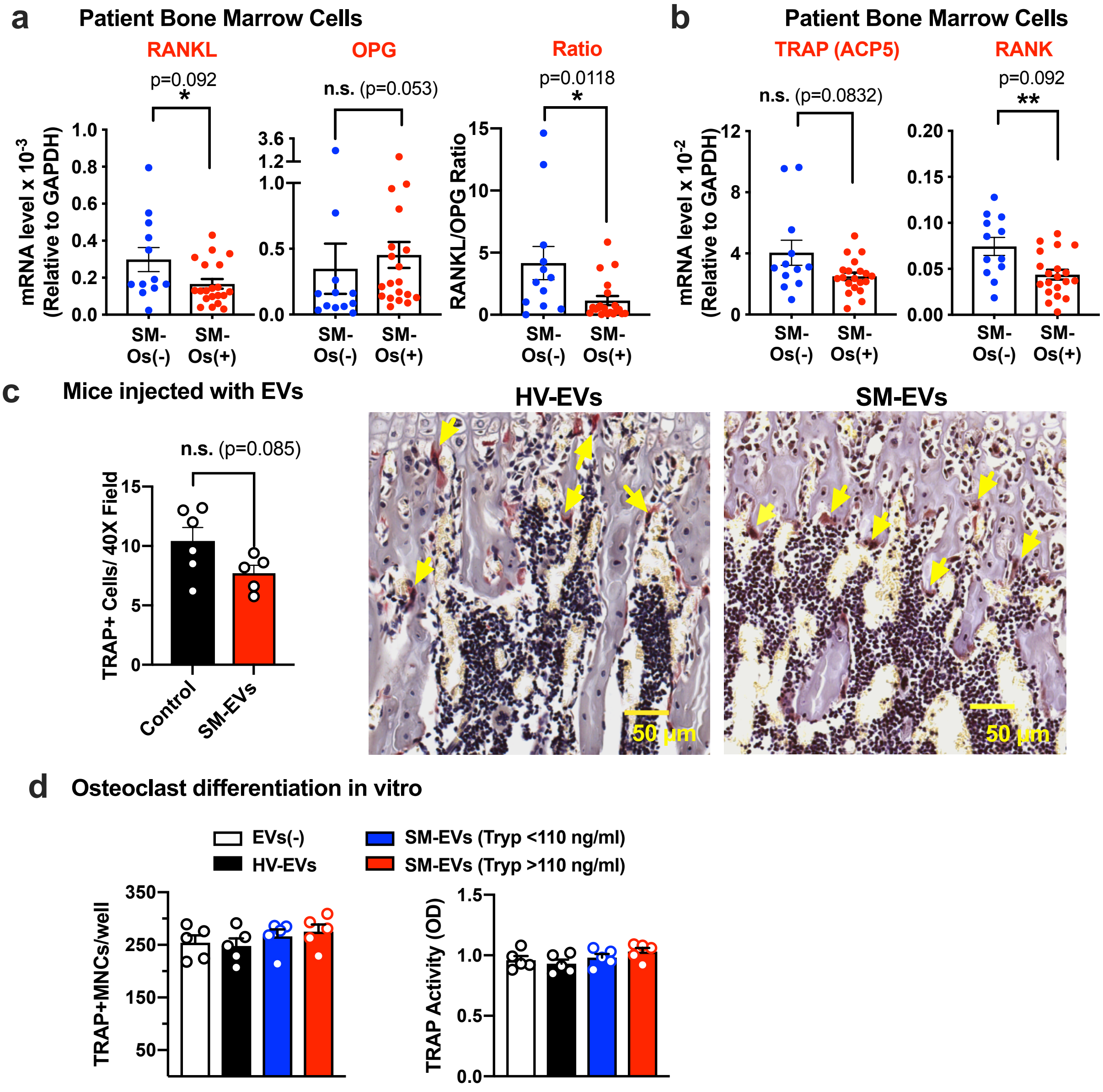

Supplementary Fig. 8-Osteoblast and osteoclast markers in bone marrow aspirates and lack of effect of SM-EVs on osteoclast differentiation. a Expression levels of RANKL and OPG mRNA and the ratio RANKL/OPG in bone marrow cells in a cohort of patients with systemic mastocytosis (SM) and osteopenia/osteoporosis (SM-Os(+)) (n=20 patient marrows) or SM without osteopenia/osteoporosis (SM-Os(-)) (n=12 patients) (see Group 3 of Supplementary Table 1). **b** Expression levels of TRAP and RANK, which are expressed by osteoclasts, in the same cohort as in **a:** SM-Os(-) (blue circles; n=12 patients) and SM-Os(+) (n=20 patients). In **a** and **b**, * $p<0.05$, ** $p<0.01$ using a Mann-Whitney test (two-tailed) and data represent the mean ± SEM. **c** Quantification of TRAP positive multinucleated cells per field of decalcified tibias in mice injected with HV-EVs (n=6 mice) or SM-EVs (n=5 mice) as

in the scheme shown in Fig. 8c. Data are the mean ± SEM. Indicated p value was obtained using an unpaired Student t-tests (two-tailed). On the right, representative images of TRAP staining in the indicated group of injected mice. Arrows indicate positively stained cells. **d** Osteoclast differentiation from mouse bone marrow macrophages for 4 days in the presence of RANKL and M-CSF, with or without 100 µg EVs from HV or patients with SM. TRAP positive multinuclear cells (MNC) per well were counted (left panel) and TRAP activity measured (right panel). Data are the mean ± SEM (n=5 biological replicates) of a representative experiment.

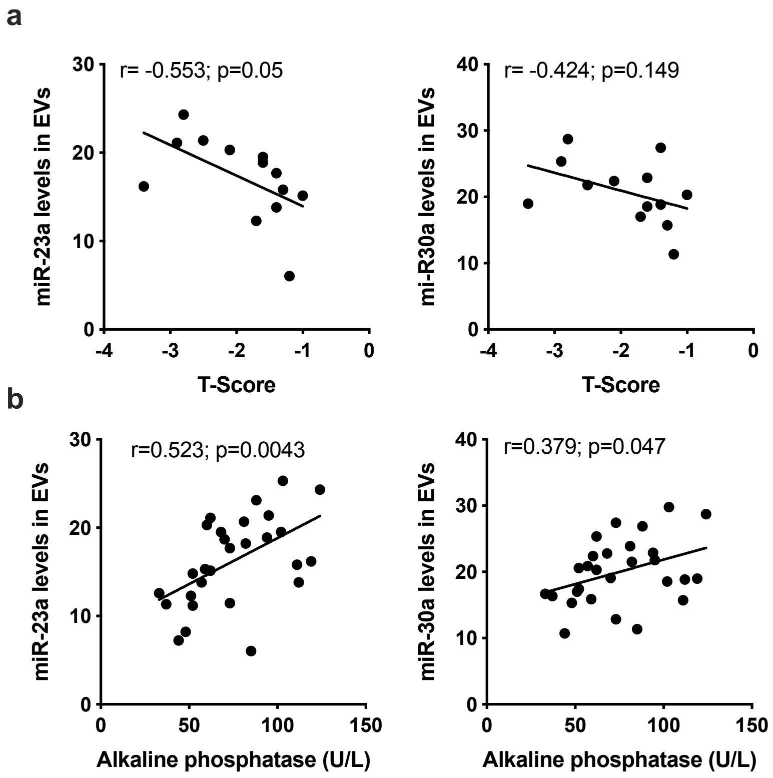

Supplementary Fig. 9- Correlations between miRNA levels in SM-EVs and clinical T-scores and serum alkaline phosphatase levels. a Pearson's correlation between the lowest T-Score in DEX scans from 1 to 3 bone sites, as indicated in the methods section, and miR-23a (left) and miR-30a (right) levels in extracellular vesicles isolated from the serum of patients with systemic mastocytosis (SM-EVs) and bone disease (n=13). One patient was excluded as the subject was the only one under Gleevec treatment. **b** Pearson's correlation between total alkaline phosphatase levels in blood and the relative levels of miR-23a (left) and miR-30a (right) in SM-EVs in all patients (n=28).

Supplementary Table 1- Characteristics and demographics of patients with SM used in this study

**Group 1**			
	**Tryptase < 110 ng/mL (n=10)**	**Tryptase > 110 ng/mL (n=11)**	**Total (n=21)**
**Gender M:F (%:%)**	40:60	18:82	29:71
**Age (mean SD)**	47.9 ± 10.5	47.8 ± 9.07	47.9 ± 9.5
**Tryptase (mean ± SD)**	48.1 ± 33.6	189.7 ± 82.5	122.2 ± 5.7
**% BM MCs (mean ± SD)**	15.0 ± 7.5	36.0 ± 19.7	26.1 ± 18.3
**% D816V-KIT positive**	90%	100%	95%
**Osteoporosis/penia**	40%	80%*	67%*
**Group 2**			
	**Osteoporosis/Osteopenia negative (n=4)**	**Osteoporosis/Osteopenia positive (n=6)**	**Total (n=10)**
**Gender M:F (%:%)**	25:75	17:83	20:80
**Age (mean ± SD)**	45.0 ± 9.3	41.2 ± 10.6	43.0 ± 9.7
**Tryptase (mean ± SD)**	26.9 ± 15.6	33.3 ± 22.1	30.5 ± 18.6
**% BM MCs (mean ± SD)**	8.8± 4.3	8.5± 4.9	8.6± 4.4
**D816V-KIT positive (%)**	75%	60%	67%
**Group 3**			
	**Osteoporosis/Osteopenia negative (n=12)**	**Osteoporosis/Osteopenia positive (n=20)**	**Total (n=32)**
**Gender M:F (%:%)**	33:67	35:65	34:66
**Age (mean ± SD)**	45.8 ± 9.5	46.5 ± 8.7	46.3 ± 10.2
**Tryptase (mean ± SD)**	91.7 ± 93.5	100.7 ± 140.6	97.0 ± 124.6
**% BM MCs (mean ± SD)**	25.9 ± 20.9	16.2 ± 16.3	19.8 ± 18.3
**D816V-KIT positive (%)**	75%	95%	87%

Group 1 are the demographics and characteristics of patients used for most EV preparations and data shown in the manuscript. Group 2 are demographics and characteristics of additional patients shown in Fig. 4c and in Supplementary Fig. 2b together with those in Group 1. Group 3 describes demographics of patients of whom bone marrow cells were obtained for transcriptional studies.

Patients were initially classified as with indolent SM (ISM) according to 2008 WHO criteria[1]. Four patients within those with tryptase >100 ng/mL in Group 1 and two in Group 3 (one in the subgroup of osteopenia/osteoporosis negative and one in the osteopenia/osteoporosis negative subgroup), meet the 2017 WHO criteria for a new variant of SM: smoldering SM (SSM)[2, 3].
The % bone marrow (BM) mast cells (MCs) were determined from bone marrow biopsies. The majority of patients were White non-Hispanic except for one Hispanic of unknown race in Group 1; one African-

American in Group 2 (in the osteopenia/osteoporosis negative group); and 2 Asian in Group 3, one in each subgroup shown.

*Note: In 3 patients it was not determined whether they had or not osteoporosis/osteopenia and were excluded from the percentages and the results where these populations were noted.

Healthy volunteers had a similar median age of 48 years.

Supplementary Table 2- Sequences for miRNA mimics and inhibitors

Mature miRNA target	miRNA sequence	Product name and Cat. no. (Qiagen)
hsa-miR-21-5p	5`-UAGCUUAUCAGACUGAUGUUGA-3'	**miRNA mimic:** Syn-hsa-miR-21-5p; MSY0000076 **miRNA inhibitor:** Anti-hsa-miR-21-5p; MIN0000076
hsa-miR-23a-3p	5`-AUCACAUUGCCAGGGAUUUCC-3'	**miRNA mimic:** Syn-hsa-miR-23a-3p; MSY0000078 **miRNA inhibitor:** Anti-hsa-miR-23a-3p; MIN0000078
hsa-miR-25-3p	5`-CAUUGCACUUGUCUCGGUCUGA-3'	**miRNA mimic:** Syn-hsa-miR-25-3p; MSY0000081 **miRNA inhibitor:** Anti-hsa-miR-25-3p; MIN0000081
hsa-miR-30a-5p	5`-UGUAAACAUCCUCGACUGGAAG-3'	**miRNA mimic:** Syn-hsa-miR-30a-5p; MSY0000087 **miRNA inhibitor:** Anti-hsa-miR-30a-5p; MIN0000087
hsa-miR-93-5p	5`-CAAAGUGCUGUUCGUGCAGGUAG-3'	**miRNA mimic:** Syn-hsa-miR-93-5p; MSY0000093 **miRNA inhibitor:** Anti-hsa-miR-93-5p; MIN0000093

Supplementary Table 3- Sequences for qRT-PCR primers

Gene/miRNA name	primer sequences (human)
ALP	F: 5'-GGACATGCAGTACGAGCTGA-3'<br>R: 5'-CCACCAAATGTGAAGACGTG-3'<br>Or Unique Assay ID:qHsaCEP0053252 (BioRad)
RUNX2	F: 5'-TCTGGCCTTCCACTCTCAGT-3'<br>R: 5'-GACTGGCGGGGTGTAAGTAA-3'<br>Or Unique Assay ID:qHsaCEP0051329 (BioRad)
SMAD1	F: 5'-TTGGTTCCAAGCAGAAGGAGGTCT-3'<br>R: 5'-AACTGAGCTAAGAGGCTGTGCTGA-3'
SMAD2	F: 5'-GTCTCTTGATGGTCGTCTC-3'<br>R: 5'-GGCGGAAGTTCTGTTAGG-3'
SMAD5	F: 5'-TTGGTGGAGAGGTGTATGCGGAAT-3'<br>R: 5'-ACAGATTGAGCCAGAAGCTGAGCA-3'
SATB2	F: 5'-AGGGGCTCCCTCTCAAATAA-3'<br>R: 5'-GAGCTGCACAACGATTCAAA-3'
CXCL12	F: 5'-ATGAACGCCAAGGTCG-3'<br>R: 5'-GGGCTACAATCTGAAGGG-3'
BMP2	F: 5'-TGTATCGCAGGCACTCAGGTCA-3'<br>R: 5'-CCACTCGTTTCTGGTAGTTCTTC-3'
GAPDH	F: 5'-GGACCTGACCTGCCGTCTAGAA-3'<br>R: 5'-GGTGTCGCTGTTGAAGTCAGAG-3'<br>Or Unique Assay ID:qHsaCEP0041396 (BioRad)
OPG (TNFRSF11B)	Unique Assay ID:qHsaCEP0053512 (BioRad)
RANKL (TNFRSF11)	Unique Assay ID:qHsaCIP0030030(BioRad)
RANK (TNFRSF11A)	Unique Assay ID:qHsaCEP0052297 (BioRad)
TRAP (ACP5)	Unique Assay ID:qHsaCEP0057829 (BioRad)
**gene name**	**primer sequences (mouse)**
Alp	F: 5'-AAGGCTTCTTCTTGCTGGTG-3'<br>R: 5'-GCCTTACCCTCATGATGTCC-3'
Runx2	F: 5'-TTGACCTTTGTCCCAATGC-3'<br>R: 5'-AGGTTGGAGGCACACATAGG-3'
Smad1	F: 5'-GCTTCGTGAAGGGTTGGGG-3'<br>R: 5'-CGGATGAAATAGGATTGTGGGG-3'
Smad2	F: 5'-AACCCGAATGTGCACCATAAGAA-3'<br>R: 5'-GCGAGTCTTTGATGGGTTTACGA-3'
Smad5	F: 5'-TTGTTCAGAGTAGGAACTGCAAC-3'<br>R: 5'-GAAGCTGAGCAAACTCCTGAT-3'
Satb2	F: 5'-TGCCTCTAGCAGTCCCAGCTCC-3'<br>R: 5'-CGTTGGCGCCGTCCACCTTA-3'
Cxcl12	F: 5'-TGCATCAGTGACGGTAAACCA-3'<br>F: 5'-CACAGTTTGGAGTGTTGAGGAT-3'
Gapdh	F: 5'-AGGTCGGTGTGAACGGATTTG-3'<br>R: 5'-TGTAGACCATGTAGTTGAGGTCA-3'
hsa-miR-21-5p	QIAGEN miScript Primer Assay

	(Cat#218300-MS00009079)
hsa-miR-23a-3p	QIAGEN miScript Primer Assay<br>(Cat#218300-MS00031633)
hsa-miR-25-3p	QIAGEN miScript Primer Assay<br>(Cat#218300-MS00003227)
hsa-miR-30a-5p	QIAGEN miScript Primer Assay<br>(Cat#218300-MS00007350)
hsa-miR-93-5p	QIAGEN miScript Primer Assay<br>(Cat#218300-MS00003346)

Supplementary Table 4 - Antibody list

Antibody	Supplier (Cat.No.)	Dilution	Clonality	Host
RUNX2	EMD Millipore (#05-1478)	1:1000	Monoclonal (AS110)	Mouse
Phospho-AKT (Thr308)	Cell Signaling (#9275)	1:1000	Polyclonal	Rabbit
Phospho-p44/42 MAPK (ERK1/2) (Thr202/Tyr204)	Cell Signaling (#9101)	1:1000	Polyclonal	Rabbit
β-Actin	Sigma-Aldrich (#A2228)	1:5000	Monoclonal (AC-74)	Mouse
AKT	Cell Signaling (#9272)	1:2000	Polyclonal	Rabbit
p44/42 MAPK (ERK1/2)	Cell Signaling (#9102)	1:1000	Polyclonal	Rabbit
BMP2	Abcam (#ab14933)	1:1000	Polyclonal	Rabbit
SMAD1/5/8 (N-18)	Santa Cruz (#sc-6031-R)	1:500	Polyclonal	Rabbit
COL1	Abcam (#ab6308)	1:1000	Monoclonal (COL-1)	Mouse
OPN	Abcam (#ab8448)	1:1000	Polyclonal	Rabbit
ANXA1	Abcam (#ab214486)	1:1000	Monoclonal (EPR19342)	Rabbit
CD9	Abcam (#ab92726)	1:1000	Monoclonal (EPR2949)	Rabbit
CD63	Santa Cruz (#sc-5275)	1:500	Monoclonal (MX-49.129.5)	Mouse
ALIX	Abcam (#ab186429)	1:1000	Monoclonal (EPR15314)	Rabbit
MRGPRX2	Abcam (#ab237047)	1:1000	Polyclonal	Rabbit
IRDye®680 RD anti-mouse	LI-COR Biosciences (#926-68070)	1:20000	Polyclonal	Goat
IRDye®800 CW anti-mouse	LI-COR Biosciences (#926-32210)	1:20000	Polyclonal	Goat
IRDye®680 RD anti-rabbit	LI-COR Biosciences (#926-68071)	1:20000	Polyclonal	Goat
IRDye®800 CW anti-rabbit	LI-COR Biosciences (#926-32211)	1:20000	Polyclonal	Goat
Runx2 (for IHC)	Abcam (ab19225)	1:1000	Monoclonal (EPR14334)	Rabbit

References

1. Horny HP, *et al.* Mastocytosis. In: *WHO classification of tumours of haematopoietic and lymphoid tissues* (eds Swerdlow SH, *et al.*). 2008 edn. IARC Press (2008).

2. Horny HP, *et al.* Mastocytosis. In: *WHO classification of tumours of haematopoietic and lymphoid tissues* (eds Swerdlow SH, *et al.*). 2017 edn. IARC Press (2017).

3. Valent P, Akin C, Metcalfe DD. Mastocytosis: 2016 updated WHO classification and novel emerging treatment concepts. *Blood* **129**, 1420-1427 (2017).
