# Supplementary material for: Mastocytosis-derived extracellular vesicles deliver miR-23a and miR-30a into pre-osteoblasts and prevent osteoblastogenesis and bone formation
Source: Nat Commun. 2021 May 5;12:2527. doi: 10.1038/s41467-021-22754-4 (PMC8100305; doi:10.1038/s41467-021-22754-4)
Supplement: Supplementary file 2 — Reporting Summary [file 41467_2021_22754_MOESM2_ESM.pdf]

nature research

Corresponding author(s): Ana Olivera

Last updated by author(s): Jan 15, 2021

Reporting Summary

Nature Research wishes to improve the reproducibility of the work that we publish. This form provides structure for consistency and transparency in reporting. For further information on Nature Research policies, see Authors & Referees and the Editorial Policy Checklist .

Statistics

For all statistical analyses, confirm that the following items are present in the figure legend, table legend, main text, or Methods section.

n/a	Confirmed	
☐	☒	The exact sample size (*n*) for each experimental group/condition, given as a discrete number and unit of measurement
☐	☒	A statement on whether measurements were taken from distinct samples or whether the same sample was measured repeatedly
☐	☒	The statistical test(s) used AND whether they are one- or two-sided<br>*Only common tests should be described solely by name; describe more complex techniques in the Methods section.*
☒	☐	A description of all covariates tested
☒	☐	A description of any assumptions or corrections, such as tests of normality and adjustment for multiple comparisons
☐	☒	A full description of the statistical parameters including central tendency (e.g. means) or other basic estimates (e.g. regression coefficient) AND variation (e.g. standard deviation) or associated estimates of uncertainty (e.g. confidence intervals)
☐	☒	For null hypothesis testing, the test statistic (e.g. *F*, *t*, *r*) with confidence intervals, effect sizes, degrees of freedom and *P* value noted<br>*Give P values as exact values whenever suitable.*
☒	☐	For Bayesian analysis, information on the choice of priors and Markov chain Monte Carlo settings
☒	☐	For hierarchical and complex designs, identification of the appropriate level for tests and full reporting of outcomes
☒	☐	Estimates of effect sizes (e.g. Cohen's *d*, Pearson's *r*), indicating how they were calculated

Our web collection on statistics for biologists contains articles on many of the points above.

Software and code

Policy information about availability of computer code

Data collection	Western blot images were obtained using Li-Cor Odyssey infrared blot imager<br>Extracellular vesicle number and size distribution by Nanoparticle tracking analysis (NTA) using a NanoSight NS300 system (NanoSight)<br>Measurements of Absorbance were performed using a plate reader (Spectra max 340PC), with a SoftMax Pro 6.2<br>Bone scan for Micro-CT was performed using a Bruker Micro CT analyzer Skyscan1172<br>Quantitative PCR was performed using a BioRad CFX96 Real-time System
Data analysis	GraphPad Prism 8 (v 8.1.1) for statistical analysis<br>For CT analysis we used SkyScan CT-Analyzer software suite (v1.15.4.0.)<br>For quantification of blots we used Image Studio (v5.2)<br>For analysis of real time PCR we used BioRad CFX Manager (v3.1)<br>Halo imaging analysis software (v3.1.1076.423)

For manuscripts utilizing custom algorithms or software that are central to the research but not yet described in published literature, software must be made available to editors/reviewers. We strongly encourage code deposition in a community repository (e.g. GitHub). See the Nature Research guidelines for submitting code & software for further information.

Data

Policy information about availability of data

All manuscripts must include a data availability statement. This statement should provide the following information, where applicable:

- Accession codes, unique identifiers, or web links for publicly available datasets
- A list of figures that have associated raw data
- A description of any restrictions on data availability

All data supporting the findings of this study are available within the main manuscript and the supplementary files or provided upon reasonable request. Raw data and original gel images corresponding to all the main and supplementary figures are included in the Source Data file.

Field-specific reporting

Please select the one below that is the best fit for your research. If you are not sure, read the appropriate sections before making your selection.

☒ Life sciences ☐ Behavioural & social sciences ☐ Ecological, evolutionary & environmental sciences

For a reference copy of the document with all sections, see nature.com/documents/nr-reporting-summary-flat.pdf

Life sciences study design

All studies must disclose on these points even when the disclosure is negative.

Sample size

For each experiment with osteoblasts, five separate wells were plated for each condition (n=5 biological replicates) , and the results obtained in the biological replicates were of high fidelity. Each experiment (with the various treatments) was repeated at least two additional times, with very similar results when comparing the different conditions. This is stated in the legends to figures and in the Statistical section in Methods. No statistical methods were used to predetermine sample size. The number of biological replicates per experiment were decided based on the literature and prior experience in our laboratory, and were sufficient to provide strong statistical significance between treatments (where observed).

For isolation of extracellular vesicles from patient serum samples we used 10-11 individuals per group, 21 patients mostly with indolent SM (divided into 2 groups of 10 and 11 respectively each, depending on their tryptase levels) and 10 healthy subjects, as indicated in Supplementary Table 1, group 1. Extracellular vesicles were also isolated from 10 additional patients with ISM to confirm the effects on osteoblasts. Given that SM is a rare disease, a total of 21 to 31 samples represent a sizable cohort. No statistical methods were used to predetermine sample size. Sample size was based on patient availability and on prior experience with extracellular vesicle isolation from patients and the reproducibility of the method.
Due to the complexity of the experiments, only in several instances each sample was tested individually, while in most experiments, vesicles from individuals within a group were pooled to test their effects on osteoblasts for a better representation of the effects . This is indicated in the legends to figures or the results section.

For mouse studies, no statistical methods were used to predetermine sample size. Sample sizes were decided based on the literature (see examples below) and prior experience. For microCT, due to the cost and limited availability of the imaging facility, we show data for a total of 7-8 separate mice per group. In Figure 9b (microCT), for simplification purposes, data from mice injected with PBS or with EVs isolated from control individuals were combined (although they are noted with distinct symbols in the figure) as these groups were not significantly different and were both considered control samples; similarly, data from mice injected with vesicles from SM patients with high tryptase or SM with osteoporosis were combined in a single group (and noted with distinct symbols in the figure) as they were also not statistically different and represent similar and overlapping populations of patients. Examples of similar sample sizes (11<n> 5) on microCT studies in mice include:
1- Chen, H., Hu, B., Lv, X. et al. Prostaglandin E2 mediates sensory nerve regulation of bone homeostasis. Nat Commun 10, 181 (2019). https://doi.org/10.1038/s41467-018-08097-7
2- Jiang, M., Liu, R., Liu, L. et al. Identification of osteogenic progenitor cell-targeted peptides that augment bone formation. Nat Commun 11, 4278 (2020). https://doi.org/10.1038/s41467-020-17417-9
3- Yu, M., Malik Tyagi, A., Li, JY. et al. PTH induces bone loss via microbial-dependent expansion of intestinal TNF+ T cells and Th17 cells. Nat Commun 11, 468 (2020). https://doi.org/10.1038/s41467-019-14148-4
4- Bok, S., Shin, D.Y., Yallowitz, A.R. et al. MEKK2 mediates aberrant ERK activation in neurofibromatosis type I. Nat Commun 11, 5704 (2020). https://doi.org/10.1038/s41467-020-19555-6.

An additional example of injection of exosomes in mice and microCT studies using n=5 mice:
5-Faict, S., Muller, J., De Veirman, K. et al. Exosomes play a role in multiple myeloma bone disease and tumor development by targeting osteoclasts and osteoblasts. Blood Cancer Journal 8, 105 (2018). https://doi.org/10.1038/s41408-018-0139-7

Data exclusions

Data were generally not excluded from the analyses.

Replication

All experiments were performed independently and successfully at least 3 times with very similar results using newly isolated extracellular vesicles each time from the same patient's populations.

Randomization	Mice were randomly assigned to groups. All had similar weight and appearance.<br>Cells were treated randomly with the different types of extracellular vesicles
Blinding	Most of the studies in vitro in osteoblasts were not blinded. Blinding of test samples was not deemed applicable because measurements were empirical and not subjective. Samples were processed identically and were reproducible.<br>Effects of bone marrow EVs on osteoblast differentiation were blinded, and histomorphology of the bone was evaluated by a pathologist blindly. For the microCT analysis the samples were numbered without knowing the exact group they were coming from.

Reporting for specific materials, systems and methods

We require information from authors about some types of materials, experimental systems and methods used in many studies. Here, indicate whether each material, system or method listed is relevant to your study. If you are not sure if a list item applies to your research, read the appropriate section before selecting a response.

Materials & experimental systems

n/a	Involved in the study
☐	☒ Antibodies
☐	☒ Eukaryotic cell lines
☒	☐ Palaeontology
☐	☒ Animals and other organisms
☐	☒ Human research participants
☒	☐ Clinical data

Methods

n/a	Involved in the study
☒	☐ ChIP-seq
☒	☐ Flow cytometry
☒	☐ MRI-based neuroimaging

Antibodies

Antibodies used	Specific information about all the antibodies used in this study is presented in Supplementary Table 4 and is as follows:<br>RUNX2, EMD Millipore (#05-1478), clone (AS110)<br>Phospho-AKT (Thr308),Cell Signaling (#9275)<br>Phospho-p44/42 MAPK (ERK1/2) (Thr202/Tyr204), Cell Signaling (#9101)<br>β-Actin,Sigma-Aldrich (#A2228), clone (AC-74)<br>AKT, Cell Signaling (#9272)<br>p44/42 MAPK (ERK1/2), Cell Signaling (#9102)<br>BMP2, Abcam (#ab14933)<br>SMAD1/5/8, Santa Cruz (#sc-6031-R)<br>COL1, Abcam (#ab6308), clone (COL-1)<br>OPN, Abcam (#ab8448)<br>ANXA1, Abcam (#ab214486), clone (EPR19342)<br>CD9, Abcam (#ab92726), clone (EPR2949)<br>CD63, Santa Cruz (#sc-5275), clone (MX-49.129.5)<br>ALIX, Abcam (#ab186429), clone (EPR15314)<br>MRGPRX2-Abcam (#ab237047)<br>IRDye®680 RD goat anti-mouse, LI-COR Biosciences, (#926-68070)<br>IRDye®800 CW goat anti-mouse, LI-COR Biosciences (#926-32210)<br>IRDye®680 RD goat anti-rabbit, LI-COR Biosciences (#926-68071)<br>IRDye®800 CW goat anti-rabbit, LI-COR Biosciences, (#926-32211)<br>Runx2 (for IHC), Abcam (#ab19225), clone (EPR14334)
Validation	The antibodies are from commercial sources and validated by suppliers for the reactivity species (human and/or mouse) and application (western blot or IHC) and by previous publications. For the following antibodies, where the supplier did not clearly specify whether they were validated, validation is shown by previous publications:<br><br>Phospho-AKT (Thr308),Cell Signaling (#9275)- The antibody registry (http://antibodyregistry.org/AB_329828) indicates applications of WB, IP, and species: hamster, mouse, rat, human, h, m, r, hm. Literature:<br>PMID:23715867, PMID:23959936, PMID:24456163, PMID:24797633, PMID:24926825, PMID:24932806, PMID:25607895, PMID:26196542, PMID:26248217, PMID:27787197, as examples<br><br>Phospho-p44/42 MAPK (ERK1/2) (Thr202/Tyr204), Cell Signaling (#9101)- The antibody registry (http://antibodyregistry.org/AB_331646) indicates applications of WB, IP, IF-IC, F for various species. Literature: PMID:16871540, PMID:18196540, PMID:18459137, PMID:19937707, PMID:23959936, PMID:25057190, PMID:25164669, PMID:25164676, PMID:25209287, PMID:25392500, as examples.<br><br>β-Actin,Sigma-Aldrich (#A2228)-Antibody registry (http://antibodyregistry.org/AB_476697) indicates vendor recommendations for WB, IC for multiple species. Literature: PMID:23546600, PMID:24280059, PMID:24654784, PMID:24949660, PMID:25368549,

PMID:25378159, PMID:25562614, PMID:25830704, PMID:26207343, PMID:26301516, PMID:26910308, PMID:27145004, as examples

For SMAD1/5/8, Santa Cruz (#sc-6031-R)- Literature:PMID:23782946, PMID:26302112, PMID:26393302, PMID:28977600, PMID:29429925, PMID:29750278, PMID:30953749, PMID:31210269, PMID:31965149, as examples.

CD63, Santa Cruz (#sc-5275), clone (MX-49.129.5)-Literature: PMID:29257951, PMID:30293865, PMID:30556910, PMID:30770248, PMID:31242427, PMID:31775218, as examples

Eukaryotic cell lines

Policy information about cell lines

Cell line source(s)	HEK293 cells and hFOB1.19 were from the ATCC collection; HMC1.1 and HMC1.2 were obtained from Dr Butterfield as referenced in Methods
Authentication	HMC1.1 and HMC1.2 are regularly used in the lab and identified by the presence of different mutations in KIT: G560V in HMC1.1; and G560V and D816V in HMC1.2.<br>The hFOB1.19 and HEK293 were certified and screed by ATCC. For hFOB1.19, upon growth at the permissive temperature, the cells differentiated as expected into osteoblasts (determined by Runx2 and alkaline phoshatase expression, production of calcium minerals and morphological changes, all shown within the results section)
Mycoplasma contamination	Certificate of analysis of hFOB1.19 cells (Batch number 63791506) was provided by ATCC and was mycoplasma free. Although we didn't have the specific batch fro HEK293 to find the certificate of analysis, ATCC thoroughly tests all cell cultures produced at ATCC before distribution to ensure that the cultures are free of mycoplasma contamination. Furthermore, all cells in the lab are randomly tested from time to time for mycoplasma since cultures of primary mast cells (handled in the same biosafety cabinets) are always ongoing in the lab and they are highly sensitive to mycoplasma
Commonly misidentified lines<br>(See ICLAC register)	No commonly misidentified cell lines were used in this study.

Animals and other organisms

Policy information about studies involving animals; ARRIVE guidelines recommended for reporting animal research

Laboratory animals	C57/B6 mice were from Jackson laboratories. Mice were 5-week old females at the time of injection (about 7-8 weeks at the time of euthanasia). For osteoclast differentiation, naive mice were 7 weeks old. The animals were shipped the NIAID (NIH) facilities, where they were housed according with the Office of Animal Care and Use guidelines under the NIH/NIAID Animal Care and Use Committee-approved animal study proposal (LAD2E), as explained in Methods
Wild animals	The study did not involve wild animals.
Field-collected samples	The study did not involve field-collected samples.
Ethics oversight	NIH/NIAID Animal Care and Use Committee (animal study proposal LAD2E)

Note that full information on the approval of the study protocol must also be provided in the manuscript.

Human research participants

Policy information about studies involving human research participants

Population characteristics	The patient population we focused on this paper include more than 31 adult patients mostly with indolent systemic mastocytosis (ISM), which is by far the category where the majority of patients with mastocytosis fall (see Supplementary Table 1 for specifics on the population). Patients with ISM generally live a normal life span, and thus suffer greatly from the long-term effects of the increased mast cell burden on the skeletal system. As to the other categories, isolated cutaneous mastocytosis is unusual in adults. The remainder of patients fall within the aggressive mastocytosis categories where there are often additional mutations, an associated hematologic neoplasm and requirement for cytoreductive agents, all of which would confound any studies on the effect of mast cells on the skeletal system. Thus, we believe this study on the effects of mast cells on the skeletal system focusing mostly on ISM is appropriate and the number of patients sufficient. Our patients were diagnosed in accordance with the World Health Organization (WHO) 2008 guidelines, and 10 adult healthy volunteers (HV) were enrolled and consented on approved protocols of the NIAID, or the Department of Transfusion Medicine (NIH). Updated WHO classifications define a new category of SM, smoldering SM (SSM), that were previously included within patients with ISM. These patients have less favorable prognosis than those in ISM but more favorable than in patients with aggressive SM. Our cohort contained 4 patients with SSM in Supplementary Table 1, Group a, and 2 in Supplementary Table 1, Group 3. Healthy volunteers had a similar median age of 48 years. These subjects were in good health and non-allergic .<br><br>The majority of patients were White non-Hispanic except for one Hispanic of unknown race in Group 1; one African-American in Group 2 (in the osteopenia/osteoporosis negative group); and 2 Asian in Group 3, one in each subgroup shown.<br><br>The characteristics of the cohort used for preparations of EVs, as indicated in the text, is shown in Supplementary Table 1 Group 1 (n=21; mean average 47.9; with 29% males and 71% females) and Group 2 (n=10; mean average 43; with 20% males and 80%

females). For RNA expression in bone marrow, additional patients (n=32; mean average 46.3; with 34% males and 66% females) were used and their characteristics summarized in Supplementary Table 1 Group 3.

In part of the study (Supplementary Table 1, Group 1), patients were divided into two subgroups: those with tryptase values below and those above the median tryptase level (110 ng/mL) in this cohort. In other instances (Group 2 and 3), patients were grouped according to the diagnosis of osteoporosis or osteopenia. Osteoporosis and osteopenia were diagnosed using a dual energy x-ray absorptiometry (DEXA) scan. According to WHO guidelines, patients with a T-score < -2.5 were recognized as having osteoporosis, and those with a T-score between -1.0 and -2.5 as osteopenia, at either the femoral neck, total femur, or AP spine.

Recruitment

Patients were recruited and diagnosed in accordance with the World Health Organization (WHO) 2008 guidelines. Stored samples were selected based on tryptase values (a minor criteria in mastocytosis) to include a wide distribution of values. For fresh bone marrows to isolate EVs, we obtained bone aspirates according to our protocol when required for clinical purposes. The first aspirates were for clinical measurements.

There were not self-selection biases as samples were chosen based on availability and tryptase values and mostly included patients with ISM for the reasons indicated above. Our cohort had generally a higher proportion of females, and although as shown in the manuscript, no differences in miRNA 23a and 30a in EVs , or number of EVs were found between females and males, further investigation on how other contents of mast cell-shed EVs (related to age, gender, or disease severity) may influence the bone environment are warranted.

Ethics oversight

Patients were enrolled and consented on protocol 02-I-0277 approved by the National Institute of Allergy and Infectious Diseases (NIAID, NIH) Institutional Review Board in agreement with the declaration of Helsinki. Fresh BM aspirates to obtain EVs as requested by one reviewer, were obtained when scheduled as determined by current ongoing protocols (02-I-0277). Samples from de-identified healthy volunteers (HV) were obtained through the NIH Department of Transfusion Medicine under the IRB-approved NIH protocol (99-CC-0168). All subjects under the protocols used in this study (and described above) gave informed consent.

Note that full information on the approval of the study protocol must also be provided in the manuscript.
